# Using Kane’s framework to build an assessment tool for undergraduate medical student’s clinical competency with point of care ultrasound

**DOI:** 10.1186/s12909-023-04030-9

**Published:** 2023-01-19

**Authors:** Gillian Sheppard, Kerry-Lynn Williams, Brian Metcalfe, Marcia Clark, Mark Bromley, Paul Pageau, Michael Woo, Yanqing Yi, Augustine Joshua Devasahayam, Adam Dubrowski

**Affiliations:** 1 Discipline of Emergency Medicine, Faculty of Medicine, Memorial University of Newfoundland, St. John’s, NL Canada; 2 Division of Orthopedic Surgery, University of Calgary, Calgary, Canada; 3 Division of Emergency Medicine, University of Calgary, Calgary, Canada; 4 Department of Emergency Medicine, University of Ottawa and Ottawa Hospital Research Institute, Ottawa, Canada; 5 Division of Community Health and Humanities, Faculty of Medicine, Memorial University of Newfoundland, St. John’s, NL Canada; 6 Toronto Rehabilitation Institute, University Health Network, University of Toronto, Toronto, Canada; 7 Faculty of Health Sciences, Ontario Technology University, Oshawa, Canada

**Keywords:** Point of care ultrasound, Medical education, Kane’s framework, Assessment

## Abstract

**Introduction:**

Point-of-care ultrasonography (POCUS) is a portable imaging technology used in clinical settings. There is a need for valid tools to assess clinical competency in POCUS in medical students. The primary aim of this study was to use Kane’s framework to evaluate an interpretation-use argument (IUA) for an undergraduate POCUS assessment tool.

**Methods:**

Participants from Memorial University of Newfoundland, the University of Calgary, and the University of Ottawa were recruited between 2014 and 2018. A total of 86 participants and seven expert raters were recruited. The participants performed abdominal, sub-xiphoid cardiac, and aorta POCUS scans on a volunteer patient after watching an instruction video. The participant-generated POCUS images were assessed by the raters using a checklist and a global rating scale. Kane’s framework was used to determine validity evidence for the scoring inference. Fleiss’ kappa was used to measure agreement between seven raters on five questions that reflected clinical competence. The descriptive comments collected from the raters were systematically coded and analyzed.

**Results:**

The overall agreement between the seven raters on five questions on clinical competency ranged from fair to moderate (κ = 0.32 to 0.55). The themes from the qualitative data were poor image generation and interpretation (22%), items not applicable (20%), poor audio and video quality (20%), poor probe handling (10%), and participant did not verbalize findings (14%).

**Conclusion:**

The POCUS assessment tool requires further modification and testing prior before it can be used for reliable undergraduate POCUS assessment.

**Supplementary Information:**

The online version contains supplementary material available at 10.1186/s12909-023-04030-9.

## Background

Point of Care Ultrasonography (POCUS) is portable ultrasound technology that is used to diagnose medical conditions and guide procedures at the bedside [1]. In Canada over the past 20 years, emergency physicians and other specialists have used POCUS to diagnose medical conditions like pericardial effusion and first trimester pregnancy [2–4]. POCUS has been shown to improve patient safety for medical procedures by improving operator effectiveness and reducing procedure-related complications [5, 6]. Given the clinical utility of this technology, competence in POCUS has been integrated into the Entrustable Professional Activities of the Royal College of Physicians and Surgeons of Canada postgraduate Competence by Design frameworks for several residency training programs [7–9].

As practicing physicians and postgraduate trainees become proficient with POCUS, educators have introduced POCUS into undergraduate medical education, with many universities describing their programs in the literature [10–22]. In 2014, 50% of the 13 accredited medical schools who responded to a survey about POCUS education in Canada had already integrated POCUS into their undergraduate medical education curriculum [23]. Although there is not a universally accepted or standardized POCUS undergraduate curriculum, several groups have published recommendations on curriculum content for undergraduate medical schools [24, 25]. Critics of POCUS in undergraduate medical education report four dominant discursive rationales repeatedly used to make claims to support POCUS for undergraduate medical education [26]. The four dominant discursive rationales are; that ultrasound allows medical students to see inside a living body and leads to a better understanding of anatomy; that ultrasound improves medical students’ ability to learn physical examination techniques; that ultrasound improves medical students’ diagnostic accuracy; and finally that undergraduate ultrasound training ensures a minimum ultrasound skill level, improving patient safety and allowing for advanced training during residency. The authors who described these four dominant discursive rationales point to the small volume and low quality of evidence available to support the ability of POCUS to improve medical students’ knowledge of anatomy or clinical skills. However, there is emerging evidence that POCUS does improve student performance on physical exam skills for standardized clinical skills assessments, improves the diagnostic accuracy for abdominal aortic aneurysm, and improves assessment scores for students who are learning the clinical exam of the abdomen [27–29].

Successful and safe integration of POCUS skills into clinical practice relies on competent end-users, particularly in the age of competency based medical education (CBME) [30, 31]. Presently, there are no validated assessment tools for POCUS skill assessment at the undergraduate level. Valid assessments are critical so that those using them can trust the results and test scores are often used to support claims that go beyond the observed performances [32, 33]. Kane’s framework describes how to generate interpretation use arguments (IUA) to make the reasoning inherent in the proposed interpretations and uses of scores explicit so that they can be better evaluated and understood. Kane’s framework for validation includes four domains: scoring, generalization, extrapolation, and implication. The scoring domain includes an observed performance with an observed score. In the case of POCUS, the observation is a student performing an observed POCUS skill which is rated and results in an observed score. The scores that are generated by the raters will be interpreted using a mean and standard deviation to determine the passing grade. Based on how a student scores on a POCUS assessment educators may make a generalization about the student’s ability to perform other POCUS clinical assessments or the quality of the POCUS curriculum. They may then go one step further and extrapolate that the students who perform well on undergraduate POCUS assessments are able to perform POCUS independently in the clinical setting. If educators are to make these generalizations and extrapolations about POCUS skills, they need to ensure that the assessment tools and scores that are used are both accurate and reliable. Building evidence for the initial scoring IUA is critical to building validity evidence in other domains for undergraduate POCUS programs.

The aim of this study was to use Kane’s framework to test an IUA for the domain of scoring for clinical competence in POCUS. The IUA in this study is that the test scores between the expert raters will be in agreement when they use a previously developed POCUS assessment tool to score participants who perform ultrasound of the heart, aorta, and abdomen [34]. To test this hypothesis, we designed and conducted a multi-center mixed methods study to determine the inter-rater reliability of a POCUS assessment tool.

## Methods

### Ethics

Ethics approval was granted by the Health Research Ethics board at the University of Ottawa (2017 0803), and the Health Research Ethics Board at the University of Calgary (REB16–1083).

### Participant selection

A convenience sample of undergraduate students, postgraduate medical trainees, and emergency medicine physicians were invited to participate in this study at three institutions, Memorial University, University of Ottawa, and the University of Calgary. Inclusion criteria for the study were: undergraduate medical students with or without POCUS training, postgraduate trainees in emergency medicine with POCUS training, or practicing emergency physicians with POCUS training. Participants were recruited at POCUS courses, scanning nights (organized sessions where students scan volunteers or standardized patients as they work toward competence in POCUS), and from local Emergency Departments. Demographic information was recorded for each participant, including their age and sex, level of medical training, whether they completed a formal POCUS course, their independent practitioner (IP) status from the Canadian Point of Care Ultrasound Society (CPOCUS) or equivalent, and the number of supervised POCUS scans they had obtained [35].

The participants were sorted into novice POCUS users or experienced POCUS users based on the number of supervised abdominal, aorta and cardiac ultrasound scans they had acquired prior to the study. These numbers were chosen based on the CPOCUS training model which assesses competency after participants have completed 50 scans per anatomical area [35]. The novice participants were defined as having fewer than 150 supervised scans. The experienced participants were defined as having > 150 supervised scans and/or IP status. To perform subsequent subgroup analyses, the participants were sorted into groups based on the level of medical training (undergraduate students/postgraduate trainees/practicing physicians).

### Expert reviewer selection

Emergency physicians who were experts in POCUS were recruited to rate the participants. The expert physician raters were asked for demographic information including their age, the number of years of experience they had using POCUS, the numbers of years of clinical practice, whether they had IP status, and the type of medical practice (generalist/specialist).

### Data collection and materials

A detailed setup and protocol to simultaneously capture the user-generated ultrasound images and the volunteer patient-scanner encounters was developed and is available in Additional file 1 Appendix A. Prior to scanning a volunteer patient, the participants were given standardized instruction or watched a one-minute video detailing the de-identification measures and expectations for the scans. The participants donned a gown, hat, and gloves to provide a measure of standardization and assist with post-processing digital anonymization. Novice ultrasound users were also offered the opportunity to watch a series of four introductory ultrasound videos. The participants and the ultrasound images were then recorded while the participant performed the following POCUS scans on a volunteer patient: abdominal ultrasound, subxiphoid cardiac ultrasound and aorta ultrasound.

The video files were edited to blur faces and the video files were coded. The coded video files were randomized and distributed to seven expert reviewers along with the assessment tool. The assessment tool had three checklists with 11 abdomen, 11 cardiac, and 9 aorta items, and a global rating scale (GRS) with 9 items [34]. The expert raters scored the videos using the checklists and the GRS and provided written comments about their overall experience using the tool and reviewing the videos. The comments from the expert raters were analyzed to understand their experience using the assessment tool. The comments were de-identified, anonymized, and loaded into a Microsoft Excel® spreadsheet, and two reviewers (AJ and GS) coded the comments separately. Using a constant comparison technique, the reviewers met twice to make sure that the themes were aligned. Once no new themes were identified, the results were analyzed.

### Data analysis

In consideration of multiple testing and the small sample size (*n* = 59), Fleiss’ Kappa (κ) statistic and the corresponding *p*-values were calculated for five items from the checklists that reflected clinical competence (focused abdominal exam Q6,8,9, Cardiac Q9, Aorta Q7) using Stata software (version 14.0; Stata Corporation, College Station, TX). The five items were selected before analyzing the data.

The Fleiss’ Kappa (κ) results were interpreted as follows: values < 0 as poor, and 0.00–0.20 as slight, 0.21–0.40 as fair, 0.41–0.60 as moderate, 0.61–0.80 as substantial, and 0.81–1.00 as almost perfect agreement [36, 37]. The Weighted Fleiss’ Kappa (κ) values were reported for all eight GRS items.

## Results

Between 2014 and 2018, a total of 86 participants were recruited: 30 participants were recruited from Memorial University of Newfoundland, 34 from the University of Ottawa, and 22 from the University of Calgary [Table 1]. Eighteen out of the fifty-nine participants included in the analysis were female and 41 were male. Twenty-one participants were undergraduate students, 26 were resident physicians and 12 participants were practicing physicians. Participants were between 21 and 57 years old. Thirty-nine participants were considered novice POCUS users and 20 participants were considered experienced POCUS users. The novice group included all the undergraduate students (*n* = 19) and some postgraduate trainees (*n* = 20). The experienced group was composed of both postgraduate trainees (*n* = 11) and physicians in practice (*n* = 9). Among the experienced group, 16 participants had IP status while four did not have IP status. Thirty-six participants had completed a formal POCUS course and eighteen had IP status with the CPOCUS [28]. Videos for 27 participants were excluded due to poor video quality, incomplete documentation, and breach of protocol, leaving 59 videos eligible for analysis.

**Table 1:** Characteristics of Participants (*N* = 59)

Characteristics	% (Number)
**Gender**	
Female	30.5 (18)
**Age**	
20–30	64 (38)
30–40	31 (18)
40–50	3 (2)
50–60	2 (1)
**Level of Medical Training**	
Undergraduate Student	32 (19)
Postgraduate Physician	52 (31)
Practicing Physician	15 (9)
**Formal POCUS Course**	
Yes	61 (36)
No	39 (23)
**Independent Practitioner CPOCUS**	
Yes	27 (18)
No	69 (41)
**Number of supervised scans**	
< 150 (novice)	66 (39)
> 150 (experienced)	34 (20)

*POCUS* point of care ultrasound, *CPOCUS* Canadian point of care ultrasound society

The characteristics of the expert raters are described in Table 2. On average, the physician raters were 45 years old, had 10 of years of experience using POCUS, and 15 years of experience practicing emergency medicine.

**Table 2:** Characteristics of Physician Raters (*N* = 7)

Variables	Mean	Range
Age, years	45	39–58
Years of POCUS practice	10	7–20
Years of clinical practice	15	5–32
IP Certification	100%	N/A
Type of medical practice		N/A
Generalist	3	
Specialist	4	

*POCUS* Point of care ultrasound, *IP* Independent Practitioner of ultrasound with CPOCUS or equivalent certification

The level of agreement between seven expert raters for all participants on five questions on clinical competency using Fleiss’ kappa ranged from fair to moderate (Abdomen Q 6: 0.52, CI 0.29–0.75; Abdomen Q 8:0.55, CI 0.33–0.77; Abdomen Q 9: 0.32, CI 0.07–0.57; Cardiac Q 9: 0.54, CI 0.32–0.77; and Aorta Q 7: 0.48, CI 0.24–0.73) (*n* = 59) [Table 3], where the confidence intervals (CI) are 95% CIs. Except Abdomen Q 9, the lower bounds of those intervals are in the range of fair agreement. After adjusting for multiple testing using Bonferroni’s correction, agreements for the four questions on clinical competency (Abdomen Q 6 and Q 8; Cardiac Q 9; and Aorta Q 7) were statistically significant (*p* < 0.01) except Abdomen Q 9 (*p* = 0.013) at the family wise error rate of 0.05. An exploratory analysis of Fleiss’ kappa for all the checklist items and the weighted Fleiss’ kappa for the global rating scale are available in Additional file 2 Appendix B.

**Table 3:** Fleiss’ kappa (κ) values for the five checklist items

Checklist Items	POCUS Users (*n* = 59)	Novices (*n* = 39)	Experienced (*n* = 20)	UG Novice (*n* = 19)	PG Novice (*n* = 20)	Experienced PG (*n* = 11)	Experienced Staff (*n* = 9)
Abdomen Q 6 Scans through the hepato-renal interface, including liver tip	0.52	0.69	0.12	0.60	0.23	−0.04	0.31
Abdomen Q 8 Scans through the spleno-renal interface	0.55	0.69	0.15	0.22	0.69	0.21	0.0
Abdomen Q 9 Identifies and sweeps the spleno-diaphragmatic interface	0.32	0.53	−0.08	0.31	0.21	0.27	−0.47
Cardiac Q 9 Identifies the pericardium	0.54	0.63	0.4	0.77	0.34	0.13	0.77
Aorta Q 7 Scans aorta from diaphragm to bifurcation	0.48	0.53	0.17	0.46	0.14	0.62	−0.20

*POCUS* point of care ultrasound, *Q* question, *UG* Undergraduate, *PG* Postgraduate

Three subgroups were assessed for rater agreement in the following categories: novice vs. experienced POCUS users, novice POCUS users who were undergraduate students vs. postgraduate students, and experienced POCUS users who were postgraduate students vs. practicing physicians [Table 3]. The agreement between seven raters for the novice and experienced POCUS users ranged from 0.53 to 0.69 (*n* = 39) and − 0.08 to 0.17 (*n* = 20) respectively. The agreement between seven raters for the novice POCUS users who were either undergraduate students or postgraduate students ranged from 0.22 to 0.77 (*n* = 19) and 0.14 to 0.69 (*n* = 20) respectively. The agreement between seven raters for the experienced POCUS users who were either postgraduate students or practicing physicians ranged from − 0.04 to 0.62 (*n* = 11) and − 0.47 to 0.77 (*n* = 9) respectively. The agreement between seven raters for all participants on 8 items included in the GRS ranged from slight to fair (0.12 to 0.31) (*n* = 12 to 59) [Supplementary Material – Additional file 2 Appendix B].

**Fig. 1:**
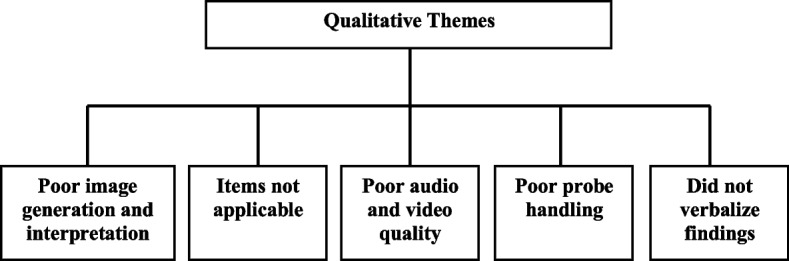
Qualitative themes that emerged from comments by the expert raters

The following themes emerged from the coded expert rater written comments: poor image generation and interpretation (22%), items not applicable (20%), poor audio and video quality (20%), poor probe handling (10%), and participant did not verbalize findings (14%) [Table 4, Fig. 1]. For items not applicable, the most common checklist items that were not applicable were focused abdominal exam item 10, cardiac item 11, and on the GRS items 5, 6, and 7.

**Table 4:** Qualitative responses from the expert raters

Themes	Total number of coded responses	Percentage of coded responses	Selected responses from the raters
**Poor image generation and interpretation skills**	34	22%	“… very uncertain probe handling and image generation” “… Needs more practice, I didn’t see a good spleno-diaphragm interface + subxiphoid cardiac scan needs work, good skills but not yet competent.”
**Poor audio and/or video quality**	30	20%	“US images don’t match the doctor-patient images!!! Patient was situ inversus? The participant says, “I’m looking at liver, hepatorenal interface, BUT he is looking in LUQ but the image is liver (in LUQ) and spleen (in RUQ)”.” “Audio poor - can’t tell but the participant demonstrated them (apex/septum/ventricles/pericardium)”
**Poor probe handling skills**	15	10%	“Improper probe orientation (FAST) …” “Probe backwards (FAST). Saw everything just backwards (FAST). Probe backwards / depth (cardiac).”
**Did not verbalize findings**	22	14%	“Did not mention (items 7/8/9 cardiac). Did not mention (item 8 aorta). Did not verbalize multiple steps.” “Not mentioned (items 7/8/9 cardiac). Clearly comfortable but did not verbalize the images.”

## Discussion

Kane’s framework is centred on stages of argument and includes scoring, generalization, extrapolation, and inference [33]. Based on Kane’s framework, our interpretation-use argument for this study was that if an undergraduate medical student performs well on a series of POCUS skill checklists and a global rating scale, they are considered competent to perform these select POCUS skills. This interpretation-use argument is based on several assumptions. First, a passing score means that an undergraduate medical student can perform the POCUS exam correctly on a patient. Second, that the physical set-up for the POCUS assessment is consistent. Third, that the raters understand how to use the assessment tool and that their scores are reproducible. Finally, that the scores would relate to real-world performance.

There was fair to moderate inter-rater reliability for the questions related to clinical competence on the three checklists (abdomen, cardiac, and aorta). In a previous systematic review of checklists and global rating scales used in the health professions, most demonstrated good inter-rater reliability with κ of 0.80 [38, 39]. Overall our checklists performed moderately with κ values between 0.33 and 0.55. Within the subgroup analysis, there was moderate agreement amongst the expert raters for novice POCUS participants. Interestingly, the expert rater agreement was poor for the more skilled clinicians using POCUS. It is possible that the novice POCUS participants may have demonstrated a clear lack of POCUS skill that was easy for the raters to distinguish through the videos. There was poor agreement amongst the raters for the other subgroups. On the GRS there was modest agreement amongst the raters for “Preparation for Procedure,” “Image Optimization,” “Probe Technique” and “Troubleshooting,” with fair to poor agreement for the other items. Overall, the scores between the raters were not reliable enough to use this scoring system to generalize, extrapolate or make inferences about undergraduate students POCUS skill level. The checklists and the global rating scale must therefore be modified and tested again before using them to assess undergraduate POCUS skills.

The strengths of this study include the large number of participants and the inclusion of multiple testing sites across Canada. In addition, we used standardized protocols and post-production video editing to provide a measure of anonymity for the volunteer patients. The expert raters were all experienced in POCUS and were from both rural and urban practice locations, with generalist and specialist training, and with a range of years of both clinical practice and POCUS experience. The raters were blinded to the level of training of the participants, and they did not have any knowledge of the scores given by the other raters. Each video was reviewed by a minimum of two raters.

There were several limitations in this study. First, there were technical issues related to poor audio and video quality. Future studies should include a set of criteria to assess the quality of the videos. Secondly, the expert raters described several problems with the scoring system. There were some items on the checklists that were not applicable to the exam. For example, there were no abnormal ultrasound findings in the volunteer patients, but there was a checkbox for identifying abnormalities and the raters were unsure how to handle this item. This may be a fault in using a checklist for this type of assessment or in the design of the tool itself which was developed by practicing physicians who use POCUS. This may be fixed in the future by giving the raters three columns to select from (yes, no, or not applicable). The expert raters did not receive any training on how to use the assessment tool. Strategies to improve the inter-rater reliability might include developing a standardized program to train the raters on the assessment tool virtually or in-person. Finally, expert-novice comparisons for validity arguments have several inherent limitations [40]. In this study, there was difficulty recruiting participants who were medical students with expert level ultrasound training, leading us to recruit postgraduate students and practicing physicians as participants. Using this tool in a heterogenous population may have negatively impacted the inter-rater reliability and the subgroup analysis explored these effects.

## Conclusion

The goal of this study was to propose and test an IUA for the scoring domain within Kane’s framework. The results of this study do not provide enough evidence to support the IUA in the scoring domain. Therefore, the POCUS assessment tool requires further modification and testing prior before it can be used for reliable undergraduate POCUS assessment.

## Supplementary Information

**Additional file 1: Appendix A.** Set-up and Protocol.

**Additional file 2: Appendix B.** An exploratory analysis of Fleiss’ kappa (κ) for all checklist items and the weighted kappa values for the global rating scale.

## Data Availability

The dataset analyzed during the current study are available from the corresponding author upon request.

## Abbreviations

POCUS: Point of care ultrasound
CBME: Competency based medical education
IUA: Interpretation use argument
CPOCUS: The Canadian Point of Care Ultrasound Society
GRS: Global rating scale
IP: Independent practitioner
