# Supplementary material for: Using Kane’s framework to build an assessment tool for undergraduate medical student’s clinical competency with point of care ultrasound
Source: BMC Med Educ. 2023 Jan 19;23:43. doi: 10.1186/s12909-023-04030-9 (PMC9854184; doi:10.1186/s12909-023-04030-9)
Supplement: Supplementary file 1 — Additional file 1: Appendix A. Set-up and Protocol. [file 12909_2023_4030_MOESM1_ESM.docx]

**Appendix A: Detailed Set-up and Protocol**

**Equipment required:**

1. AV.iO HD Epiphan USB video grabber (<http://www.epiphan.com/products/avio-hd/>)
2. VGA cable
3. USB cable
4. USB extension cables
5. 2 Webcams (we are using a Logitech Webcam C930e and a HuddleCamHD)
6. USB multiport (with 3 ports)
7. VSee Free software (Mac) (You will need two accounts, one to make the call, and another “dummy” account that you can call)
8. Iris screen capture software (Mac)

**How to start recording:**

1. Connect video grabber to ultrasound machine using an VGA cable. Connect the USB cable from the video grabber to the multiport.
2. Mount a webcam (Logitech Webcam C930e) to the screen of the ultrasound machine. Connect the USB cable from the camera to the multiport.
3. Mount the second webcam (HuddleCamHD) at an angle above the foot of the bed. Connect the USB cable from the camera to the multiport. *Note: You may require USB extension cables for this step.*
4. Connect the multiport to a laptop, and open VSee.
5. Under preferences, you will need to set up which cameras you will be using, select the video tab:
   1. Under device, set the main camera as the camera mounted on the U/S machine. Change the resolution to HD if it does not automatically select it. You do not want a mirrored feed, so ensure that it is not checked:
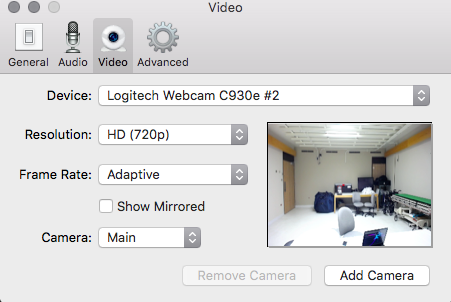

   2. Next, select Add Camera. In the camera dropdown menu, select Aux 1 and assign it to the other camera. Again, change the resolution to HD if it is not automatically selected. Ensure the show mirrored box is not selected:


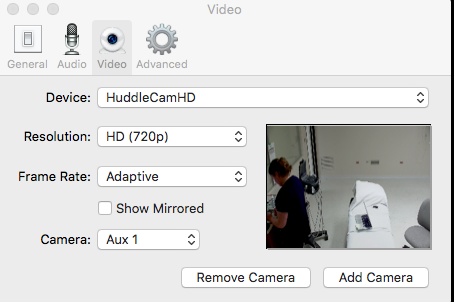


- 1. Click Add Camera a second time. Now in the camera dropdown menu, slect Aux 2 and assign it to the video grabber (U/S feed). Change the resolution to HD if it is not automatically selected:
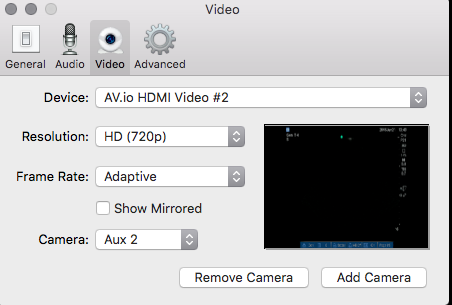

  2. Now all three video feeds are ready to go. It is important now to assign which camera is providing audio. Still under preferences, on the audio tab, select the camera mounted to the ultrasound machine as the audio input device:
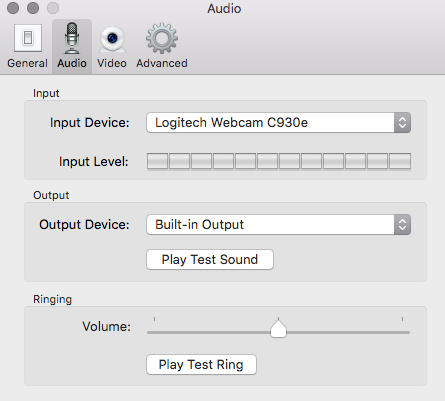


1. On your VSee screen, make a video call. All the windows from the various cameras will pop up, and they can be resized and positioned accordingly. The screen capture software will record exactly as you have it arranged on your screen:


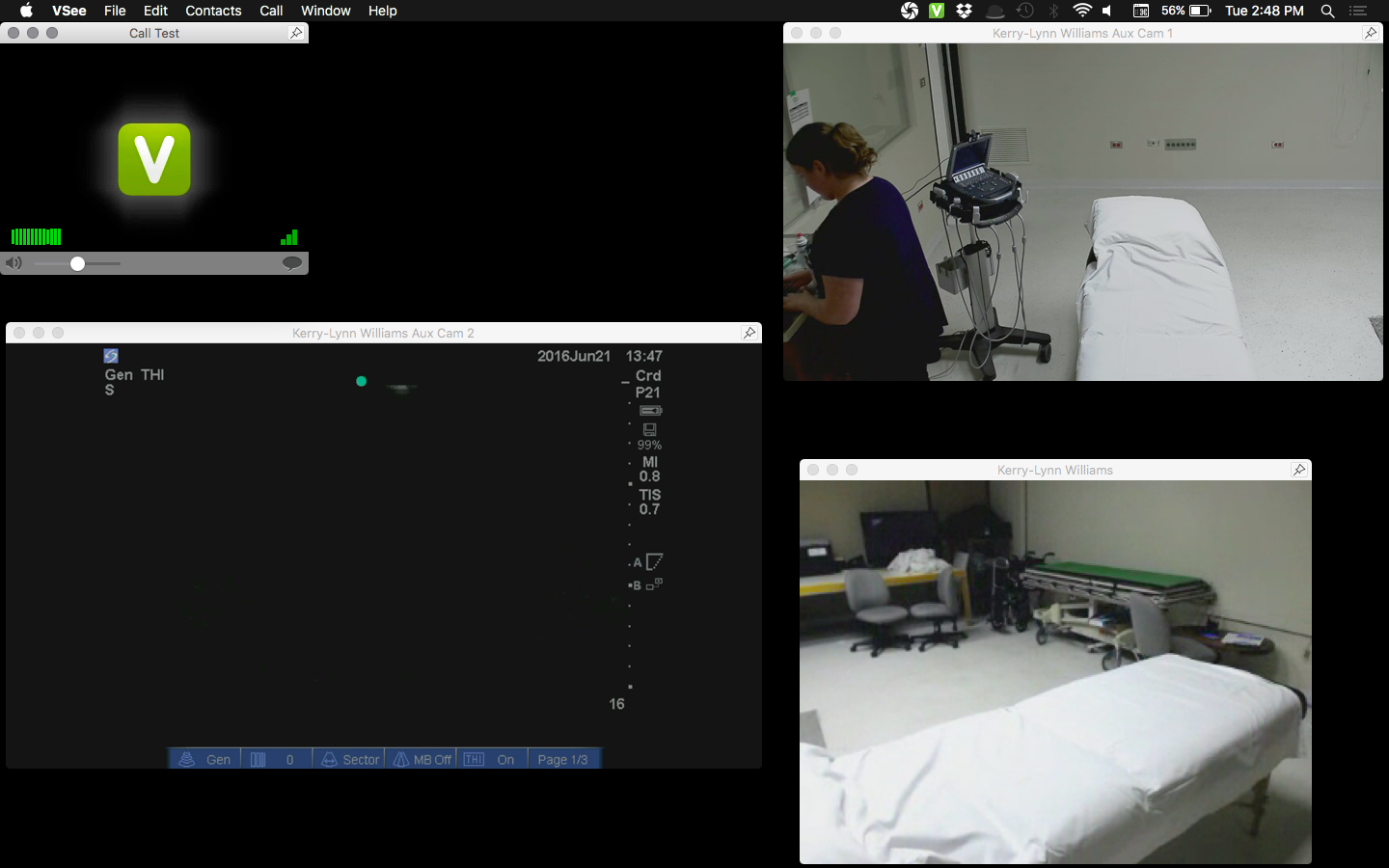


1. When you are ready, start a new recording using Iris, which opens in your taskbar:


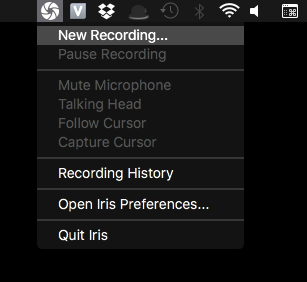


1. A box with video preferences will open on your screen. Keep the video preferences as seen below (h.264 – 30 fps and Actual Size). Change both audio preferences to Default Input, and under Annotation, select No cursor. Shown is the free version of Iris, the watermark can be removed by purchasing the software. You will not have to readjust the windows in between calls, but you will if the setup is dissembled.
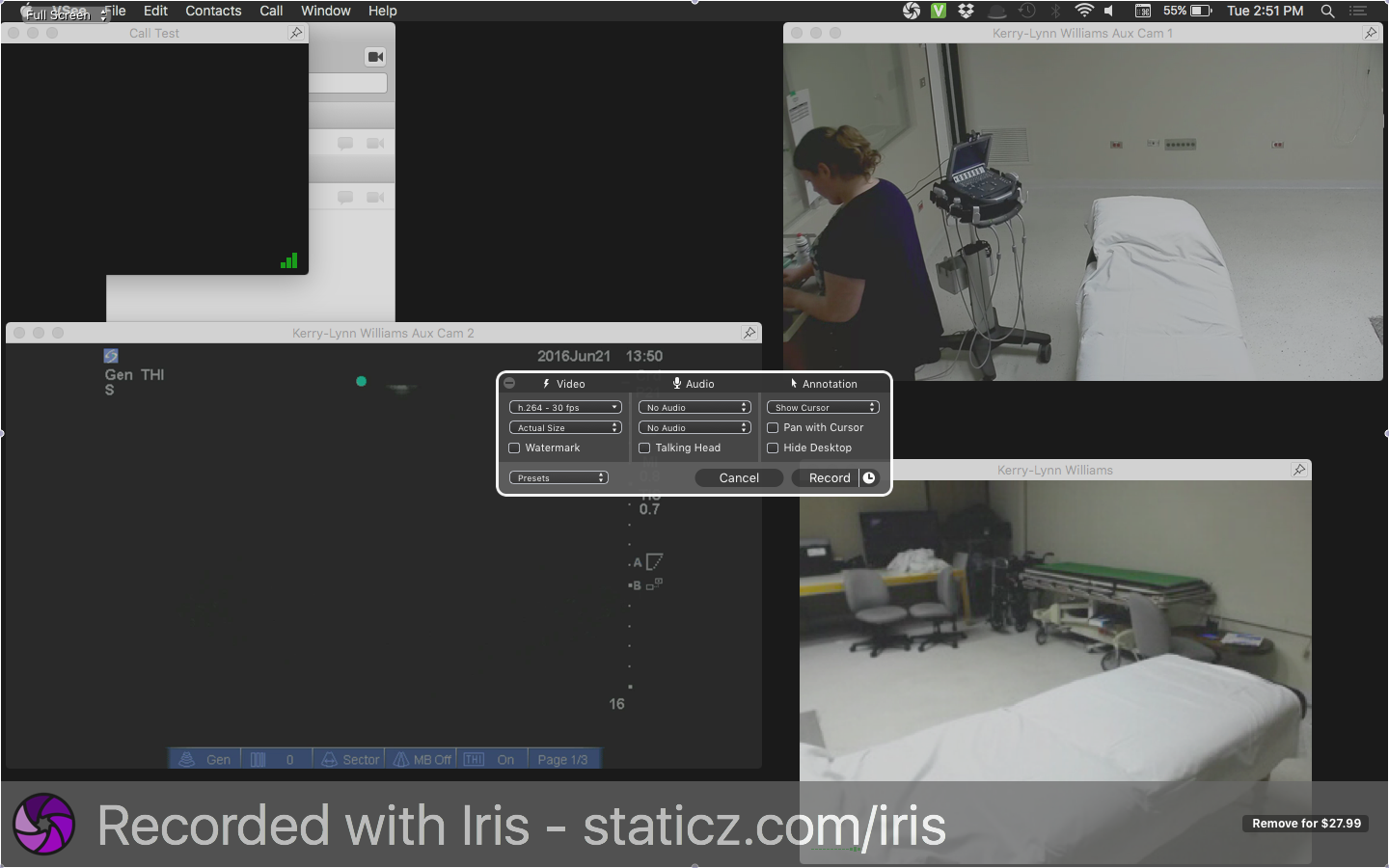


**Protocol**

**Prior to attending the session participants are offered the opportunity to watch a series of online videos if they are novice to ultrasound:**

Subxiphoid Cardiac: https://youtu.be/BEofsBzfOOw

Aorta: https://youtu.be/NI-tU5w-gzg

LUQ FAST exam: https://youtu.be/VBHCmw8iHCc

RUQ FAST exam: <http://youtu.be/lzgxZsFZhTU>

**Start**

1. Set probe choice to Linear Array (so the learner must adjust it to begin scanning).
2. Ask SP to place arms at their sides.
3. Ensure SP has gel cleaned off and gown covers abdomen to begin.
4. Ensure necessary supplies are on ultrasound cart (gel, wipes, probe cleaner, hand sanitizer).

**Scanner instructions**

1. Direct scanner to complete demographic scanner form.
2. Show scanner the introductory video.
3. Instruct scanner to approach scenario as if it were a real patient. Verbalize what they are seeing and doing, treat it like an OSCE, and remind them that there is a 10 minute time cap with a one minute warning.
4. Instruct participant not to move ultrasound machine.
5. Ensure both videos are labeled with code.
6. Record data in excel spreadsheet.
7. Ask participant to introduce themselves as a medical student (instead of using their name)
8. Ask participants to scan in the following order:
   1. RUQ/LUQ FAST (abdomen)
   2. Subxiphoid cardiac
   3. Aorta
