# Supplementary material for: Using Kane’s framework to build an assessment tool for undergraduate medical student’s clinical competency with point of care ultrasound
Source: BMC Med Educ. 2023 Jan 19;23:43. doi: 10.1186/s12909-023-04030-9 (PMC9854184; doi:10.1186/s12909-023-04030-9)
Supplement: Supplementary file 2 — Additional file 2: Appendix B. An exploratory analysis of Fleiss’ kappa (κ) for all checklist items and the weighted kappa values for the global rating scale. [file 12909_2023_4030_MOESM2_ESM.docx]

**Appendix B:** An exploratory analysis of Fleiss’ kappa (κ) for all checklist items and the weighted kappa values for the global rating scale

Exploratory Analysis of Fleiss’ kappa (κ) for all Checklist Items

|  | **Fleiss kappa (κ)** | **95% Confidence Interval** |
| --- | --- | --- |
| **Checklist Item** |  |  |
| Abdomen 1 | 0.43 | 0.17 - 0.68 |
| Abdomen 2 | 0.80 | 0.64 - 0.96 |
| Abdomen 3 | 0.49 | 0.24 - 0.74 |
| Abdomen 4 | 0.34 | 0.02 - 0.67 |
| Abdomen 5 | 0.47 | 0.020 - 0.75 |
| Abdomen 6 | 0.52 | 0.29 - 0.75 |
| Abdomen 7 | 0.70 | 0.51 - 0.90 |
| Abdomen 8 | 0.55 | 0.33 - 0.77 |
| Abdomen 9 | 0.32 | 0.07 - 0.57 |
| Abdomen 10 | N/A (n=6) | N/A |
| Abdomen 11 | 0.49 | 0.26 - 072 |
| Cardiac 1 | 0.14 | -0.13 – 0.40 |
| Cardiac 2 | 0.48 | 0.24 – 0.72 |
| Cardiac 3 | 0.28 | 0.01 – 0.56 |
| Cardiac 4 | 0.11 | -0.17 – 0.38 |
| Cardiac 5 | 0.34 | 0.07 – 0.61 |
| Cardiac 6 | 0.75 | 0.56 – 0.94 |
| Cardiac 7 | 0.38 | 0.13 – 0.62 |
| Cardiac 8 | 0.43 | 0.17 – 0.68 |
| Cardiac 9 | 0.54 | 0.31 – 0.77 |
| Cardiac 10 | 0.45 | 0.21 – 0.68 |
| Cardiac 11 | 0.38* | -0.42 -1 |
| Aorta 1 | 0.24 | -0.01 – 0.5 |
| Aorta 2 | 0.48 | 0.24 – 0.72 |
| Aorta 3 | 0.23 | -0.05 – 0.51 |
| Aorta 4 | 0.14 | -0.13 – 0.42 |
| Aorta 5 | 0.43 | 0.16 – 0.70 |
| Aorta 6 | 0.27 | 0.01 – 0.52 |
| Aorta 7 | 0.48 | 0.24 - 0.73 |
| Aorta 8 | -0.15* | -0.23 - -0.07 |
| Aorta 9 | 0.41 | 0.17 – 0.65 |

The quadratic weights are used in the calculation of the Fleiss kappa for the Global Rating Scale (GRS)

| **GRS Item*** | **Fleiss kappa (κ)** | **95% Confidence Intervals** |
| --- | --- | --- |
| GRS 1 | 0.55 | 0.34 – 0.75 |
| GRS 2 | 0.55 | 0.37 – 0.73 |
| GRS 3 | 0.63 | 0.46 – 0.81 |
| GRS 4 | 0.65 | 0.48 – 0.82 |
| GRS 5 | 0.08* | -0.47 – 0.63 |
| GRS 6 | 0.08* | -0.61 – 0.77 |
| GRS 7 | -0.05* | -0.45 – 0.35 |
| GRS 8 | 0.60 | 0.4 – 0.79 |

* The missing method in Gwet, K.L. 2014. (Handbook of Inter-Rater Reliability: The Definitive Guide to Measuring the Extent of Agreement Among Raters. 4th ed. Gaithersburg, MD: Advanced Analytics) was used.

^ The Stata package developed by Klein, D (The Stata Journal (2018) 18, Number 4, pp. 871–90 1was used.
